# The Acoustic Dimension of Reading: Does Musical Aptitude Affect Silent Reading Fluency?

**DOI:** 10.3389/fnins.2020.00399

**Published:** 2020-04-29

**Authors:** José Manuel Foncubierta, Francisco H. Machancoses, Kris Buyse, M.C. Fonseca-Mora

**Affiliations:** ^1^Education Department, Faculty of Education, Psychology and Sports Sciences, University of Huelva, Huelva, Spain; ^2^Department of Linguistics, Faculty of Arts, KU Leuven, Leuven, Belgium; ^3^Predepartamental Unit of Medicine, Science Health Faculty, Jaume I University, Castellón, Spain; ^4^Department of Applied Languages, Faculty of Languages and Education, Nebrija University, Madrid, Spain; ^5^English Studies Department, Faculty of Humanities, University of Huelva, Huelva, Spain; ^6^Center of Contemporary Thinking and Innovation for Social Development (COIDESO), University of Huelva, Huelva, Spain

**Keywords:** silent reading fluency, musical aptitude, foreign language, acoustic dimension, auditory working memory, phonological awareness, contextual word recognition, adult reader

## Abstract

Fluent reading in a foreign language includes a complex coordination process of visual and auditory nature as the reading brain transforms written symbols into speaking auditory patterns through subvocalization (inner voice). The auditory information activated for reading involves the projection of speech prosody and allows, beyond letters and words decoding, the recognition of word boundaries and the construction of the melodic contours of the phrase. On the one hand, phonological awareness and auditory working memory have been identified in the literature as relevant factors in the reading process as skilled readers keep the acoustic information in their auditory working memory to predict the construction of larger lexical units. On the other hand, we observed that the inclusion of musical aptitude as an element belonging to the acoustic dimension of the silent reading aptitude of adults learning a foreign language remains understudied. Therefore, this study examines the silent reading fluency of 117 Italian adult students of Spanish as a foreign language. Our main aim was to find a model that could show if linguistic, cognitive and musical skills influence adults’ silent reading fluency. We hypothesized that learners’ contextual word recognition ability in L1 and FL in addition to, phonological awareness, auditory working memory and musical aptitude, elements related to the acoustic dimension of reading, would influence adults’ silent reading fluency. Our structural modeling allows us to describe how these different variables interact to determine the silent reading fluency construct. In fact, the effect of musical aptitude on fluent silent reading in our model reveals to be stronger than phonological awareness or auditory working memory.

## Introduction

### The Acoustic Dimension of Reading in a Foreign Language

Either in the mother tongue (L1) or in a foreign language (FL), the reading process implies the inter-relationship between written and spoken language. Ahissar et al. (2000), for instance, studied adults’ reading abilities and concluded that auditory processing abilities accounted for more than 50% of the reading score variance, even in the group of adults who never had childhood histories of reading difficulties. Tichko and Skoe (2018) pointed out that “sensorineural auditory processing in central auditory structures is related to reading ability across the lifespan, beginning in the preliterate period and continuing into adulthood” (p.2), while Mankel and Bidelman (2018) stated that the brain’s neural encoding and perception of sound differences is simply due to inherent auditory abilities that belong to the acoustic dimension. Therefore, an appropriate acquisition of oral skills eases the processes of triggering word recognition and fluency both necessary for reading comprehension (Dehaene, 2009). In alphabetic and shallow languages, such as Spanish and Italian, phonological awareness or the identification and manipulation of units in oral language is a reliable indicator of word recognition (McBride-Chang, 1995; Share, 1995, 2008): fluent reading is not possible without efficient contextual word recognition (Wang et al., 2005; Koda, 2007a; Macalister, 2010). In this sense, although letters to sounds conversion is a critical subskill for word recognition and reading fluency, the role of phonology appears to be more complex than simply support of word-by-word visual recognition. While reading silently or aloud, the identification of words is not enough, nor is it enough considering learners’ ability of discriminating, remembering, and manipulating sounds at the sentence, word, syllable, and phoneme level, a lack of sensitivity toward the rhythmic and melodic properties of a given language also produces difficulties in accessing and comprehending a written text (D’Imperio et al., 2016). Thus, our study examines the acoustic dimension of reading. More concretely, the silent reading fluency of Italian adult students of Spanish as a foreign language in order to find a plausible model where the interaction between linguistic, cognitive and musical skills could explain adults’ silent reading fluency. We hypothesized that learners’ contextual word recognition abilities in L1 and FL in addition to phonological awareness, auditory working memory and musical aptitude, elements related to the acoustic dimension of reading, explain adults’ silent reading fluency.

As regards phonological awareness, Ashby et al. (2013) showed in a longitudinal study the relationship between phonological awareness and silent reading fluency where results of phonemic tasks done by children studying Grade 2 accounted for nearly 42% of the variance in total time during silent reading in Grade 3. These data challenge the shift hypothesis and the accounts of reading development that claim that the role of phonology in reading is minimized as fluency develops and readers access word meanings directly from the orthographic form. They concluded that phonological processing continues to contribute to the efficiency of word recognition processes even in fluent readers. Macaruso and Shankweiler (2010, p. 464–465) carried out a study to identify a set of predictors that might be useful in distinguishing between less skilled and average college students readers. A discriminant analysis showed that the best predictors were a measure of phonological awareness (spoonerism) and a measure of verbal working memory (digit span). According to their results, phonological awareness and verbal working memory were more sensitive in identifying less skilled readers in the sample. Together these two variables predicted group membership correctly for 77% of the cases.

In foreign language reading, phonological awareness is considered as a precursor of the reading ability in different languages (Koda, 2007b). Kato (2009) studied Japanese students learning English as a second language and showed that phonological processes are required in foreign language silent reading at least until the learner becomes very proficient in the second language. The results of this research evidence that highly significant correlations are maintained between the sentence processing performance when reading silently and the reading comprehension score. For proficient readers, the involvement of the orthographic skills remained significant but phonological skills were still highly necessary for low proficient language learners.

Research on silent reading has shown that readers use their inner voice to project prosodic elements (intonation, tone, stress, and rhythm) on written symbols in order to disambiguate confusing sentences, create phonic chunks and predict lexical items (Kadota, 1987; Fodor, 2002; Ashby, 2016). According to the Prosodic Structure Hypothesis (Kadota, 1987), during FL silent reading the reader’s inner voice or subvocalization follows speech rhythm patterns that support prediction of stressed syllables. This subvocalization plays an essential role when including words in syntactic and semantic relationships, allowing the reader to organize texts into lexical chunks. Even more, Ashby (2016) states that phonological decoding itself is a conscious process. The unconscious process of transforming visual information into their correlative sounds would only be possible when automatically activating the phonological word form before it is captured according to the prosodic information contained in the syllable, such as intensity, pitch and duration (phonological precoding stage). Therefore, the melodic and rhythmic structure of the text is built during contextual word recognition as well as during sentence integration, facilitating reading speed. As phonological precoding requires high-quality phonological representations of spoken words both during FL and L1 reading experience, research has been conducted into the influence of L1 orthographic and phonological coding on the FL reading ability (Sparks, 1995; Sparks et al., 2012). In this vein, transference from reading subskills like L1 phonological awareness into FL is well documented (Wang et al., 2005; Ziegler and Goswami, 2006; Bernhardt, 2010).

Unlike children, adult readers have more difficulties in distinguishing phonemic contrasts between L1 and FL (Kuhl et al., 2006). Apart from neurophysiological reasons such as the age of exposure to the foreign language (brain plasticity), in the case of FL reading fluency acquisition, the degree of phonological transfer may also be influenced by the proximity or similarity between the two languages (Ziegler and Goswami, 2006; Russak and Saiegh-Haddad, 2011; Yamashita, 2013) or by individual differences in working memory.

The second aspect of the acoustic dimension considered in our study is auditory working memory, another key concept of both reading and musical abilities (Kraus and Chandrasekaran, 2010). Baddeley et al. (1985) highlights the role that working memory plays as a component of fluent reading. Other works such as Strait et al. (2011) demonstrated the importance of auditory working memory for oral and silent reading fluency. In their study, higher auditory working memory correlated with better reading performance. Linguistic and musical information requires a temporary information storage system for their correct manipulation and integration, fundamental for reading prosody (Strait et al., 2011). To understand a phrase, the skilled reader needs to keep phonemic information in memory and integrate it in order to build lexemes and their semantic representation. In fact, reading with natural prosody facilitates sentence organization in memory and increases recall (Koriat et al., 2002). In the same way, processing melodic information requires tones to be kept in memory in order to integrate them in the melodic phrase representation. Pechmann and Mohr (1992) added the tonal loop, where prosodic and musical processing share resources of the auditory working memory.

Finally, musical aptitude, understood as a range of inherent abilities for music that an individual is born with and that are possibly shaped by informal exposition to music, has also been considered as a fundamental element of the acoustic dimension as it builds humans’ auditory abilities (Patel, 2011; Slevc, 2012; Besson et al., 2017). In fact, music and speech prosody are communication sounding systems supported by the same acoustic parameters such as frequency, duration, intensity and timbre (Chobert and Besson, 2013). Slevc and Miyake (2006) considered that “being skilled at music means having a “good ear” for perceiving and analyzing foreign speech sounds” (p. 675) and showed that “individuals who are good at analyzing, discriminating, and remembering musical stimuli are better than other people at accurately perceiving and producing L2 sounds” (p. 679). Several studies have shown evidence of musical aptitude and pronunciation of a second language, both relying on cognitive processes of the auditory working memory, where tonal and verbal memory have a similar functional architecture (Tanaka and Nakamura, 2004; Koelsch et al., 2009; Jordan, 2018). This implies an overlap of neural structures from early ages on (Christiner and Reiterer, 2018). According to Jordan (2018: 177), “both musicians and non-musicians have an additional component, such as a tonal loop, which supports the retention of tone sequences”. In other words, to some extent the brain processes speech as a kind of music (Koelsch, 2011). The effect of learners’ musical aptitude has been mainly related to FL phonological perception and production (Milovanov et al., 2010), but less clear is its connection to FL reading skills. Studies about musical aptitude and “seemingly” visual reading skills such as silent reading fluency, remain to be scarce and inconclusive (Zeromskaite, 2014; Gordon et al., 2015), especially with adult readers who learn a language in a foreign context (Swaminathan et al., 2018). Gómez-Domínguez et al. (2019) provided insights into how music perception affects early reading skills in 63 Spanish children learning English. Their findings support a transfer of music perception abilities to L1 young learners’ reading abilities that affect the alphabetic principle, the phonemic awareness and the word recognition skills in their FL early reading skills.

Studies focusing on the relationship between language perception, musical skills and reading abilities confirm the hypothesis that music and language rely on similar mechanisms of auditory temporal processing (Patel, 2011; Besson et al., 2017). Nevertheless, two issues are still debated: on the one hand, studies that argue that differences in reading abilities mediated by musical aptitude could be the result of genetic mediated differences (Schellenberg, 2015; Swaminathan and Schellenberg, 2017). On the other hand, empirical studies indicate that it is specific musical training that could exert a causal influence on the subjects’ abilities to discriminate language sounds and to get better results in reading (Kraus and Chandrasekaran, 2010; Chobert and Besson, 2013; Besson et al., 2017). There are even longitudinal studies of educational intervention that show how musical training improves language perception and reading skills (Besson et al., 2007; Flaugnacco et al., 2015). However, Bigand and Poulin-Charronnat (2006) pointed out that musical aptitude could be acquired by “musically experienced listeners” only through exposure to music without explicit musical training. Thus, being a non-musician does not mean that one does not have musical aptitude. Individuals with extensive musical training do not always reach higher levels of musical competence than those without formal musical training (Law and Zentner, 2012).

In this study, the term musical aptitude represents the music abilities of individuals with or without musical training. Our hypothesis is that musical aptitude, as a capacity measured by the participant’s Tuning, Melody, Accent and Tempo abilities, shapes the acoustic dimension of reading because fluent reading requires a sensibility toward the phonological, rhythmic and melodic properties of any language. Taking all this together, in our model we hypothesize that if “reading fluency involves every process and subskill involved in reading” (Wolf and Katzir-Cohen, 2001: 220), then silent reading fluency can be operationalized as a complex construct where different visual and oral components interact: phonological awareness, auditory working memory and L1/FL visual contextual word recognition.

Therefore, this study aims to uncover, through correlations and structural equation modeling (SEM), the acoustic dimension of silent reading fluency based on an analysis of factors such as L1 and FL contextual word segmentation, in addition to phonological awareness, auditory working memory and musical aptitude of 117 Italian university students of Spanish as a foreign language. Our research questions based on correlations are to confirm in our sample what previous research about phonological awareness, word identification and segmentation, auditory working memory and reading has already tested. Given that a lack of sensitivity toward the rhythmic and melodic properties of a given language could also produce difficulties in accessing and comprehending a written text (D’Imperio et al., 2016), our study is aimed at searching for a statistical-causal model between musical aptitude and silent reading fluency. Moreover, it is the first time to our knowledge that musical aptitude is correlated with L1 and FL word segmentation.

**FIGURE 1:**
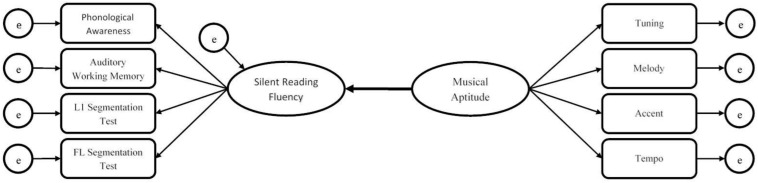
Theoretical SRF model.

The study is structured around five research questions (see Figure 1), all of them related to the silent reading fluency of adult readers:

RQ1: Is there any relationship between L1 segmentation and FL segmentation?
RQ2: Is there any relationship between phonological awareness and FL segmentation?
RQ3: Is there any relationship between auditory working memory and FL segmentation?
RQ4: Is there any relationship between musical aptitude subtests and L1/FL segmentation?
  RQ4a: Is there any relationship between musical aptitude subtests and L1 segmentation?
  RQ4b: Is there any relationship between musical aptitude subtests and FL segmentation?
RQ5: Can we establish a statistical-causal model for determining silent reading fluency on the basis of L1 and FL segmentation, phonological awareness, auditory working memory and musical aptitude?

The eight observed variables (phonological awareness, auditory working memory, L1 and FL contextual word recognition/segmentation, tuning, melody, accent and tempo) have been measured directly. From these measured variables, the latent variables (silent reading fluency and musical aptitude) are reflected if the model is true.

In order to find out how musical aptitude influences silent reading fluency as hypothesized in Figure 1, a SEM was carried out to understand if and how musical aptitude could influence silent reading fluency, and how the eight observed indicators would interact with each other and with the latent variables of this study in our sample. SEM provides a statistical method which “enables researchers to easily set up and reliably test hypothetical relationships among theoretical constructs as well as those between the constructs and their observed indicators” (Deng et al., 2018, p. 1).

These measurement components are shown in Figure 1 by using thin lines. By convention, the direction of the arrows goes from the latent variables to the observed ones.

## Materials and Methods

### Participants

Data was collected from 124 adult readers, all of them students of the University of Macerata, of whom only 117 answered all the tests. All participants were freshmen and passed a language level test called “Test di linguistic idoneitaÌ” that the university uses to classify them into a homogeneous pre-intermediate language level class. All participants belonged to the same class. Of the 117, 34.19% (*n* = 40) were male, and 65.81% (*n* = 77) were female students. Age ranged between 21 and 25 years, with an average of 21.72 (Sd = 0.771). All subjects were native speakers of Italian studying a Degree Program in Linguistic and Cultural Mediation in English and Spanish. They had never participated in any immersion program in Spain and acknowledged not suffering any kind of reading disability. Most of them had not received musical training (only 4.7% had received some training before).

### Measures

Students were administered five different tests: a contextual word recognition test in its Spanish version, a contextual word recognition test in its Italian version, a Spoonerism test to measure learners’ phonological awareness, WAIS-IV to measure learner ìs auditory working memory (Digit Span tests, Letters and Numbers Sequencing and Arithmetic) and the musical MiniProms Test in order to check their musical aptitude.

**FIGURE 2:**
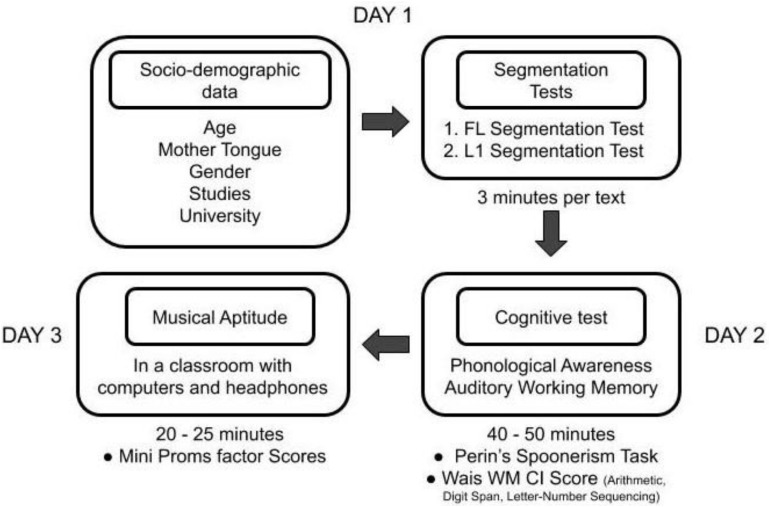
Data Collection protocol flowchart.

Figure 2 includes our data collection protocol flowchart and in the following paragraphs each test is explained.

#### L1 and FL Contextual Segmentation Tests

The Spanish and Italian contextual word recognition or in brief, L1 and FL segmentation tests, were adapted versions of the Test of Silent Contextual Reading Fluency (Hammill et al., 2006). These tests measure the participants’ level of reading fluency in each language by counting the number of printed words that could be segmented within 3 min in a text without blank spaces. The participants were presented with the text of Human Rights in its Spanish and Italian version. Both versions were based on different articles of the Universal Declaration of Human Rights in order to avoid transfer of previous knowledge. Readability tests were performed with a view to control that the selected Spanish and Italian texts fit the college level (45.6 Spanish Flesch Reading Formula, 30.17 Italian Flesch-Vacca and 44 Italian GulpEase). GulpEase index was rated similar to the Italian Flesch-Vacca adaptation but better tailored to the Italian language (Forti et al., 2019, p.360).

Letters were all in lowercase because “the lowercase letters offer the reader a skyline of words” (Hiebert and Reutzel, 2014, p. 37). In order to measure speed and correctness of word recognition in the text, participants had 3 min to recognize as many words as possible using a ballpoint pen and making separations with bars. First, they did the test with the FL text and subsequently the other text in L1. The results were obtained from the total number of correctly identified words within the fixed time period. Data collection time was 6 min.

#### Phonological Awareness

The phonological awareness test is a Spanish adaptation of Perin (1983). In the original version of this task, American famous people’s names were used; for example, “Chuck Berry.” It was administered individually. Students had to listen to 18 pairs of first and last names of famous Spanish people (for example, Peneélope Cruz [peneélope kruéθ]), and were asked to change the initial consonant of the name by the initial consonant of the surname, producing Ceneélope Pruz [θeneélope pruéθ], in such a way that [t∫eneélope pruéθ] or [keneélope pruéθ] were considered non-valid. After hearing the name, they only had 4 s to respond. An Olympus Ws-650S tape recorder was used for data collection. The data collection time was 2 min per participant.

#### Auditory Working Memory

Furthermore, participants scored individually their auditory working memory. Digit Span backward and forward, and Arithmetic of the WAIS-IV test (Wechsler et al., 2008) were administered, in addition to Letters and Number Sequencing. These subtests evaluate auditory working memory. Following the WAIS-IV test score indications, the AWM score was computed from the sum of Arithmetics, Digit Span and Letters and Numbers Sequencing, gathering the AWM Scalar Punctuation. Afterward, this score is transformed in CI scores using the scales offered by the WAIS-IV correction manual. Data collection time was approximately 30 to 35 min for each participant.

#### Musical Aptitude

Mini-PROMS, the reduced version of the Proms test (Zentner and Strauss, 2017), was administered individually, each student with a computer and headphones. This reduced version was selected due to the high number of tests and the amount of class time needed. Mini-Proms consists of a battery of subtests that measure musical aptitude through the discrimination of different musical structures, namely Tuning, Melody, Accent and Tempo. The tuning subtest plays a C-chord whose tone E could be mistuned. Participants are asked to judge whether the tuning is the same in the reference and the probe stimulus. In the melody subtest participants hear a two-bar monophonic harpsichord melody twice, followed by the probe melody which can differ slightly by one or more tones. Accent assesses the capacity of detecting and retaining rhythmic patterns in a sequence of 5 to 12 beats. The tempo subtest comprises rhythmically and timbrally diverse stimuli which are the same between reference and probe stimulus, except, potentially, for their tempo. The data collection time was 20 to 25 min.

### Data Analysis

First, a descriptive analysis of the variables has been carried out (Table 1). The normality of these variables has been tested using the Kolmogorov-Smirnov (KS) normality test. Before starting the SEM analysis, we wanted to know if there were correlations in accordance with our research questions. As mentioned earlier, our correlational questions check if our results are consistent with the ones previously reported in literature although mainly for children and referring to L1. Phonological awareness and auditory working memory have already been consistently identified as predictors of early reading ability and we wanted to check the same type of correlations with our adult population. We think this gives more support to the SEM we carried out based on our working hypotheses.

**TABLE 1:** Descriptive statistics.

	Mean	SD	Median	Min	Max	Ks (p)
L1 segmentation test	195.56	54.64	207	74	364	0.087 (0.043)
FL segmentation test	120.67	55.18	119	29	288	0.085 (0.013)
Phonological awareness	12.26	1.71	12	5	16	0.155 (0.000)
Auditory working memory	87.66	8.53	87	72	118	0.203 (0.000)
Proms melody score	7.22	3.17	7	1	15	0.115 (0.024)
Proms tuning score	6.03	2.12	6	1	16	0.124 (0.000)
Proms accent score	6.86	2.16	7	2	12	0.079 (0.048)
Proms tempo score	7.39	2.48	7	2	15	0.120 (0.000)

To determine the statistical-causal model that interrelate all variables, we conducted a SEM analysis with the Multivariate Software program EQS 6.2 (Bentler, 2008). Although there is debate about the sample size needed for SEM, we considered our sample of 117 participants suitable to perform the proposed structural modeling because correlations were strong (Kenny, 2015). In order to describe how different variables interact in the silent reading fluency construct, SEM is a better-chosen analysis technique than the classical methods of regression because it assigns dependent and independent variables to cause and effect categories, including their order of appearance. SEM provides a statistical method for evaluating relationships among indicators and latent variables in a hypothesized model, and provides causal statistical fit indices of the hypothesized model. Our structural model integrates eight directly measured variables (L1 and FL contextual word segmentation, phonological awareness, auditory working memory, tuning, melody, accent and tempo) and two multi-factorial latent variables: silent reading fluency and musical aptitude (see Figure 1, where latent variables are represented by circles and observed variables by squares, with arrows showing the relations between these variables).

When the variables did not follow a normal distribution, the robust statistic of Satorra-Bentler (Satorra and Bentler, 1988; Satorra, 1990; Yuan and Bentler, 2007) was used. This robust statistical procedure allowed us to contrast hypotheses concerning relationships among latent variables and indicators, including the different interrelations between them, when the assumptions of normality and heteroscedasticity do not occur.

The EQS also offers the Lagrange Multiplier Test, a procedure designed to study the need for constraints on the model, both the equality constraints that may have been included, and the covariance not initially included and that should be counted as free parameters (Bentler, 2008). This test is analogous to the so-called LISREL Modification Indices, with the difference that the Lagrange Test operates multivariately in determining misspecified parameters in a model, while the LISREL Modification Indices operate univariately (Byrne, 2013, p. 84). As the Lagrange Test indicated the introduction of modifications, they were tested until we reached the fitted model.

## Results

The main descriptive statistics of the variables under study, as well as the K-S test of normality, are presented in Table 1. In order to answer research questions 1 to 4, a correlational analysis using Spearman Rho (ρ) with a bilateral significance test was performed to test the relational hypothesis (Table 2), given the non-normality of the variables (*p* < 0.05).

**TABLE 2:** Spearman’s rho Correlations.

	L1ST	FLST	PA	AWM	PMS	PTS	PAS	PTmS
L1 segmentation test	1	0.750**	0.645**	0.609**	0.692**	0.656**	0.705**	0.658**
FL segmentation test		1	0.668**	0.729**	0.807**	0.615**	0.711**	0.523**
Phonological awareness			1	0.694**	0.635**	0.466**	0.641**	0.658**
Auditory working memory				1	0.781**	0.541**	0.543**	0.587**
Proms melody score					1	0.680**	0.771**	0.742**
Proms tuning score						1	0.554**	0.527**
Proms accent score							1	0.760**
Proms tempo score								1

**Correlation is significant at the 0.01 level (2-tailed).

The Spearman Rho (ρ) test reveals a highly significant relationship between L1 Segmentation and FL segmentation [RQ1] (ρ = 0.750), between FL segmentation and phonological awareness [RQ2] (ρ = 0.645), between auditory working memory and FL segmentation [RQ3] (ρ = 0.609), and between musical aptitude subtests and L1 segmentation [RQ4a] (Melody: ρ = 0.692; Tuning:0.656; Accent:0.705; Tempo:0.658). Also, there is a strong correlation between musical aptitude subtests and FL segmentation [RQ4b] (Melody: ρ = 0.807; Tuning:0.615; Accent:0.711; Tempo:0.523) (see Table 2). All these correlations have a significance *p* < 0.01.

**FIGURE 3:**
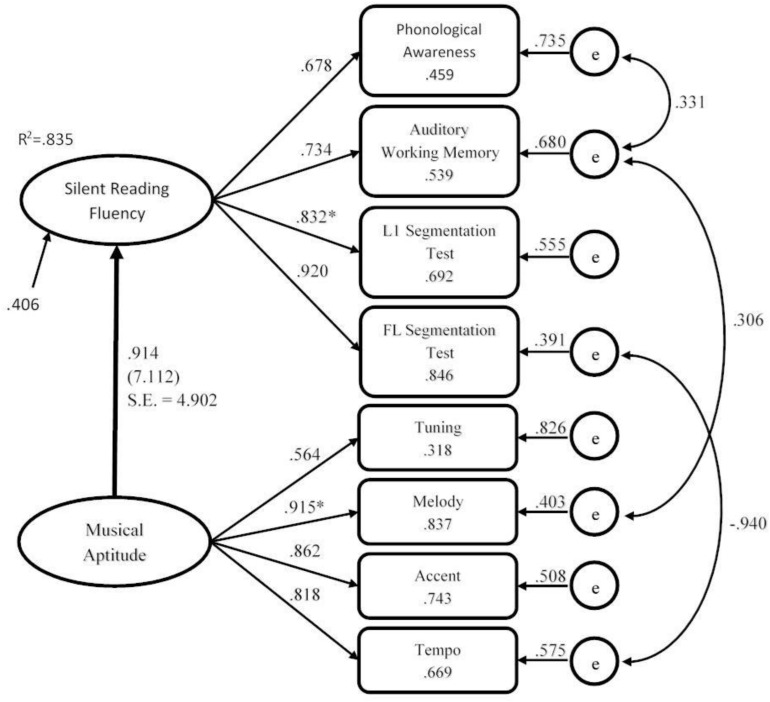
Final SEM Model obtained in standardized values. All coefficients are significant. The fixed parameters were marked with “*”. Robust Independence Model χ2 = 619.753; Satorra-Bentler Scaled χ2 = 22.601 (p = 0.093): Non-Normed Fit Index 2816 = 0.976; Comparative Fit Index = 0.987; Root Mean Square Error of Approximation [90% CI] = 0.066 [0.000, 0.118]; e = error.

In order to more comprehensively examine relationships among musical aptitude and silent reading fluency, we subjected these data to SEM in Figure 1 [RQ5]. All covariances and saturations between the variables are represented in a path diagram with their fit indexes (Figure 3). According to MacCallum et al. (1996) and Schreiber et al. (2006), RMSEA values between 0.06 and 0.08, and other coefficients greater than or equal to 0.95 indicate an appropriate fit. Therefore, considering the results obtained, we can determine that our model fits appropriately.

High saturation of musical aptitude on silent reading fluency (β = 0. 914) was observed. The latent construct musical aptitude is determined significantly by the four mentioned components: tuning (γ = 0.564), melody (γ = 0.915), accent (γ = 0.862) and tempo (γ = 0.818); and silent reading fluency is determined significantly by the indicators L1 segmentation (γ = 0.832) and FL segmentation (γ = 0.920), in addition to phonological awareness (γ = 0.678) and auditory working memory (γ = 0.734).

The inclusion of a series of covariances among the indicators, based on information provided by the Lagrange Test, helped to adjust the model. These covariances have been included through an iterative process, in which the fit of the model for each covariance introduced was tested. Especially relevant were covariances between phonological awareness and auditory working memory (φ = 0.331), and the one between auditory working memory and melody (φ = 0.306). Also, covariances between Tempo and FL segmentation (φ = −0.940) were found.

**FIGURE 4:**
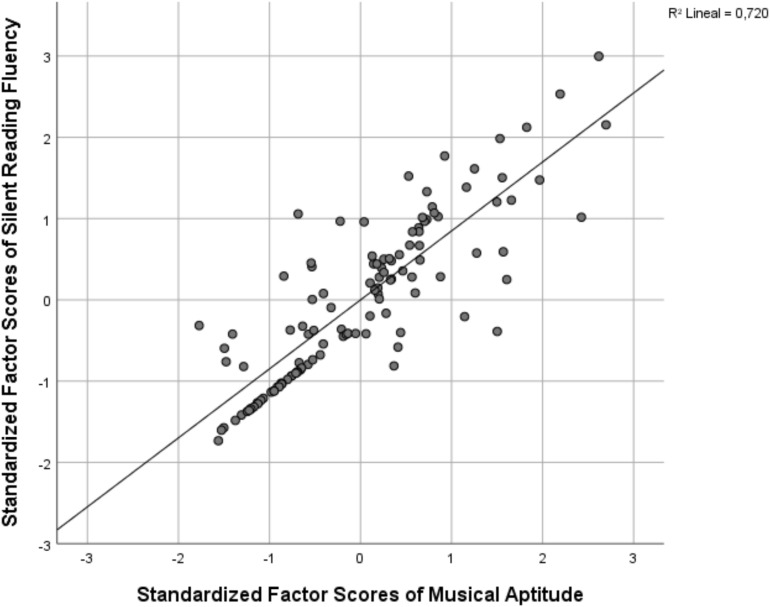
Silent reading fluency positively correlates with musical aptitude (factor scores extracted from SEM).

In order to observe the saturation between musical aptitude and silent reading fluency, a scatterplot analysis was carried out, showing a linear *R*^2^ of 0.720 between the factorial scores in standardized values obtained for each subject (Figure 4).

## Discussion

The objective of this study was to uncover the acoustic dimension of silent reading fluency based on an analysis of factors such as contextual word recognition in L1 and FL, in addition to phonological awareness, auditory working memory and musical aptitude among 117 Italian university students of Spanish as a FL. We expected that these variables could explain learners’ individual differences in their silent reading fluency. More concretely, we wanted to know if musical aptitude affects silent reading fluency. The analysis provides us with the following answers to the different research questions.

### RQ1: Is There Any Relationship Between L1 Segmentation and FL Segmentation?

Regarding the first question, a strong correlation was found between the L1 segmentation and FL segmentation. As put forward by Sparks et al. (2012) in their *Linguistic Coding Differences Hypothesis*, skills acquired in the mother tongue, such as fluent reading, can be transferred to foreign language learning. This transfer, as well as its degree (Young-Kim et al., 2017) may also be due to the proximity or similarity between orthographic codes of the two languages (Wang et al., 2005; Ziegler and Goswami, 2006; Bernhardt, 2010). In fact, in transparent languages such as Spanish and Italian, with a consistent grapheme-phoneme relationship, fluent reading develops earlier than in alphabetic languages with a more complexly decodable spelling system such as English. Nevertheless, regardless of the typological and linguistic similarity of the two languages, the contextual word recognition ability in the foreign language scored lower. In this sense, the results are consistent with earlier studies where reading in a FL occurs slower than in L1 (Koda, 2007b; Bernhardt, 2010; Yamashita, 2013). According to previous literature, this deceleration could be due to the grade of familiarity between FL and L1 but also to the learners’ accumulated reading experience in L1.

### RQ2: Is There Any Relationship Between Phonological Awareness and FL Segmentation?

The results of our analysis point out a strong correlation between both variables. Kato (2009) found out that phonological decoding plays an important role for low language proficient FL readers, at least, in two situations: while reading unfamiliar words, and when it is necessary to keep information in memory at the same time that processing complex structures. In our study, participants had a pre-intermediate level and we increased the difficulty of the silent reading fluency task by asking students to read a visually complex text, since words were not separated by blank spaces. In this way, being able to visually recognize letters, syllables and words requires to keep in memory the conversion of letters into sounds. As difficulties in phonological awareness are usually the hallmark of reading difficulty (Ziegler and Goswami, 2006; Perfetti, 2007; Russak and Saiegh-Haddad, 2011), we expected that the ability of retaining acoustic features in memory and to manipulate them was related to silent reading fluency. Phonological awareness is a construct composed of at least three components -general cognitive ability, verbal memory, and speech perception-, but a large part of phonological awareness is simple speech perception (McBride-Chang, 1995). As phonological awareness is a reliable indicator of visual word recognition in FL reading (Wang et al., 2005; Koda, 2007a), our results reveal that part of the individual differences in FL word recognition are due to the ability to perceive sounds and manipulate them in a non-native language. The proximity between the two languages also shows the strong correlation between phonological awareness and L1 word recognition ability.

### RQ3: Is There Any Relationship Between Auditory Working Memory and FL Segmentation?

The results of our study show a correlation between auditory working memory and FL segmentation, which is weaker in L1 than in FL, probably due to the learners’ greater mastery and reader confidence in their L1 (Russak and Saiegh-Haddad, 2011). We expected that the use of a text without blank spaces between words or spelling signs would force readers to mentally pronounce the words they are discovering while reading (Kadota, 2002); for that, readers need to maintain acoustic information in mind to integrate sounds into larger units and build meaning. The orthographic information without phonological decoding is purely iconic and does not allow the grouping of sound blocks according to the melodic and rhythmic pattern of the language. In this sense, silent reading fluency implies the cooperation of sound information and its corresponding meaning beyond words. As silently reading a text without spaces requires to manage the letter-sound relationship in order to recognize words, and also to integrate this information into larger units, the theoretical construct of working memory presented by Baddeley (1986) plays an essential role in discussions on the mechanisms employed in L1/FL segmentation. Especially, the component called phonological loop allows readers to manipulate and store speech-based information and is further divided into a phonological short-term store and an articulatory control process. The former is in charge of temporarily maintaining phonological information, the latter of refreshing fading phonological information through subvocal rehearsal.

However, readers also need to process melodic information from syllables (intensity, pitch, duration), in order to predict the phonological form of words and their composition spelling (Koriat et al., 2002). According to Ashby (2016), this precoding occurs automatically and requires out of the syllabic information certain prosodic elements in order to complete a word, such as when we complete a song from the beginning of its melody. This process demands, therefore, a tonal loop so that tones are kept in memory and integrated in the melodic phrase representation (Pechmann and Mohr, 1992; Tanaka and Nakamura, 2004; Jordan, 2018).

### RQ4: Is There Any Relationship Between Musical Aptitude Subtests and L1/FL Segmentation?

Our results indicate that musical aptitude subtests correlate highly and positively with L1 segmentation (RQ4a) and FL segmentation (RQ4b). To our knowledge, there are no other studies on musical aptitude and L1/FL segmentation. Previous studies such as Slevc and Miyake (2006) or Milovanov et al. (2010) had already shown a relationship between musical aptitude and FL learning, especially at the phonological level and the acquisition of other oral skills. As for the relationship between sensitivity to different musical structures (tuning, melody, accent, and tempo) and visual word recognition, our data show that musical aptitude holds a high correlation with L1 segmentation as well as with FL segmentation. Zeromskaite (2014, p.85) in a literature review claims that “the theoretical basis behind the reading skills facilitation by music is less clear, but it may be best explained by increased listening sensitivity.” In a meta-analysis by Gordon et al. (2015), only a weak trend was found toward significance of musical discrimination abilities on reading fluency. They hypothesize that music skills share more variance with phonological skills (due to their auditory bases) than with reading fluency skills (more visual skills), and thus music training may have larger effects on phonological awareness than on reading. Nevertheless, our results point out that likely adult readers’ musical aptitude is affecting their contextual word recognition ability.

### RQ5: Can We Establish a Statistical Causal Model for Determining Silent Reading Fluency on the Basis of L1 and FL Segmentation, Phonological Awareness, Auditory Working Memory and Musical Aptitude?

In order to find out how musical aptitude affects silent reading fluency, a SEM was carried out (see Figure 3). The results allowed us to test our model proposed in Figure 1. We included three *post hoc* modifications. The Lagrange Test for computing parameters recommended us to add covariances between Auditory Working Memory and Phonological Awareness, Auditory Working Memory and Melody, and FL Segmentation Test and Tempo. All covariances and saturations between the variables are represented in a path diagram with their fit indexes (Figure 3).

The theoretical approach is highly relevant when trying to present a new model. So, when the test indicated these possible covariances between auditory working memory and melody, and in order to improve the fit of the model, we first checked whether they had a prior theoretical justification for adding them and we found the following support for the inclusion of these covariances. The use of covariance to fit the model is not conventional, but authors such as Byrne (2013, p. 184) point out that it is reasonable to use it when the theoretical basis supports it. Kline (2015, p. 380) states that “the capability to explicitly model the error covariance structure is an advantage of SEM over more traditional statistical techniques.”

The covariance between the values of phonological awareness and auditory working memory shows that differences in silent reading fluency are also determined by the retention capacity of acoustic elements such as phonemes for word recognition, as pointed out in the Baddeley (1986) working-memory model that includes the phonological loop. Regarding the integration of information in the oral reconstruction of reading, the covariance between auditory working memory and melody may indicate that the ability to retain musical information, such as the succession of single tones, could be related to the reading intonation which is necessary to understand a text, as this intonation is also present in students’ silent reading. This recognition of tonal frequencies points to the importance of tonal memory in the development of silent reading fluency (Pechmann and Mohr, 1992; Tanaka and Nakamura, 2004).

On the other hand, the results are consistent with previous studies that show how melody is the main musical feature affecting phonological awareness in adult readers (Posedel et al., 2012; Kempe et al., 2015). The covariance between phonological awareness and auditory working memory and between auditory working memory and melody, may reflect that tasks used for both variables (phonological awareness and melody), have in common the same cognitive processing demand which is the temporary information storage system required for their correct manipulation (Strait et al., 2011).

Similar studies carried out with children while reading aloud show that rhythm-related skills often predict phonological awareness (Tierney and Kraus, 2014). Nevertheless, as put forward by Swaminathan and Schellenberg (2017, p. 1930), among adult readers “the story is more complicated.” Likely, adult readers are more experienced listeners than children.

As stated by Koelsch (2011), to some extent the brain processes speech as a kind of music, but when learning a foreign language some musical features of the mother tongue may remain. This seems to be the case of the negative covariance between tempo and FL segmentation. This covariance, known as negative transference (Melby-Lervåg and Lervåg, 2014), due to the proximity or similarity between the two languages, could indicate the influence of an individual characteristic of the L1 rhythmic pattern. As Italian is characterized by the elongation or duration of the accented vowels, this value may show that Italian learners of Spanish are using their Italian rhythmic patterns, which goes in line with the interference hypothesis of the L1 rhythmic pattern (Iversen et al., 2008). Their central idea is that depending on the L1 musical features, there is a certain influence on the perception of non-linguistic musical traits, hence that negative covariance influences FL and not L1. In theory, the Italians would perceive tempo differences better in Italian than in Spanish as it is a characteristic of their L1. In Italian, tonic vowels receive a greater emphasis on duration than Spanish tonic vowels. This would mean that duration is a relevant phonological aspect in Italian but not in Spanish, where the duration does not produce a change in meaning in the system; that is, it would only have a pragmatic value: when a speaker extends the duration of a vowel to add a connotative meaning. The negative covariance with (only) FL segmentation would be an example of negative transfer in FL reading: with less musical tempo, more FL silent reading fluency. The high value of the covariance between tempo and FL segmentation would not indicate that they are identical variables, but they may mean that for Italian language learners of Spanish the ease of recognizing a musical aspect such as tempo is inversely proportional to their ability of segmenting a text in Spanish.

Taken all these data together, it can be argued that the high saturation of musical aptitude on silent reading fluency confirms that the ability of perceiving the differences of tuning and tempo along with accent and melody may contribute more to the understanding of the individual differences in silent reading fluency than other factors.

## Conclusion

The general conclusions of this study allow us to consider that the musical aptitude of adult readers studying a foreign language gives shape to their reading skills. Other cognitive components involved in reading such as the auditory working memory appear to be fundamental to the integration of linguistic and musical information, playing a crucial role in explaining the individual differences in silent reading fluency. To some extent, we expected that the correlational study could yield positive results. Earlier studies had already reported the positive correlations between reading skills in L1 and second languages (Koda, 2007a; Gómez-Domínguez et al., 2019), or between phonological awareness and reading components such as fluency (van den Boer et al., 2014; Flaugnacco et al., 2015), but we decided to check it again to present our model. The SEM, as a statistical-causal method, allowed us to analyze how variables would behave after previously observed correlations, according to an *a priori* hypothesized model.

The many significant results may be also due to the nature of our research design where all tests represent a demand on participants’ auditory working memory. The L1 and FL segmentation tests involve reading a complex text with no blanks, in which readers need to retain the sequences that they are recoding in their memory. The phonological awareness test requires keeping sounds in memory in order to manipulate them, and the musical aptitude test also calls for the retention of auditory information. Although further research is still needed, the level of significance found in our results may reveal the existence of common cognitive and neural mechanisms for language reading and musical skills, so that readers with better results in the musical aptitude, segmentation, and the phonological awareness tests are also demonstrating a better ability in the task of maintaining information in their auditory working memory.

Given the novelty of our vision on how musical aptitude explains adult readers’ silent reading fluency, it still requires further study especially with other foreign languages and other adult populations. Our model based on the acoustic dimension of silent reading fluency offers an image about the interaction of visual and sound factors related to reading. In agreement with Grabe and Stoller (2011), readers are extraordinary word recognizers and, moreover, according to our data, good readers are excellent melody recognizers and this affects their silent reading fluency.

## Data Availability Statement

The datasets generated for this study are available on request to the corresponding author.

## Ethics Statement

Ethical review and approval was not required for the study on human participants in accordance with the local legislation and institutional requirements. The patients/participants provided their written informed consent to participate in this study.

## Author Contributions

MF-M contributed to the conceptualization, investigation, and funding acquisition. FM contributed to the methodology and formal analysis. JF wrote the original draft. JF, FM, KB and MF-M wrote, reviewed and edited the manuscript.

## Conflict of Interest

The authors declare that the research was conducted in the absence of any commercial or financial relationships that could be construed as a potential conflict of interest.
